# Supplementary material for: Developmental and genetic modulation of evidence integration dynamics in zebrafish sensorimotor decision-making
Source: Commun Biol. 2026 Oct 29;9:1435. doi: 10.1038/s42003-026-11264-y (PMC13723567; doi:10.1038/s42003-026-11264-y)
Supplement: Supplementary file 1 [file 42003_2026_11264_MOESM1_ESM.pdf]

nature portfolio

Peer Review File

Developmental and genetic modulation of evidence integration dynamics in zebrafish sensorimotor decision-making

Corresponding Author: Professor Armin Bahl

This file contains all reviewer reports in order by version, followed by all author rebuttals in order by version.

Version 0:

Reviewer comments:

Reviewer #1

(Remarks to the Author)
In this manuscript, Garza et al. apply a drift-diffusion framework to early larval sensorimotor responses. Their model extracts individual-level information from freely swimming larvae and further examines how these parameters change across development and in disease-associated mutants.

This is a very interesting manuscript that is likely to be of broad interest to both the zebrafish and neuroscience communities. The study is conceptually strong, and the approach is innovative. A few modifications could further strengthen the manuscript. They provide a solid framework with sound methotology and statistical analysis.

Major comments
• Given the broad scope of the journal, additional background on some of the core concepts would be helpful. In particular, the section discussing Bayesian optimization could be expanded to clarify why this approach was selected over alternative methods. Similarly, the Introduction may be easier to follow if the section beginning around line 55 were moved before line 46. Drift-diffusion is first introduced on line 46, but the manuscript does not yet provide sufficient context for non-expert readers on what this framework is and why it is useful in this setting.

• The developmental experiment tracking behaviour across time is interesting. However, other studies, including Roberts et al (2017), Cleal et al (2023), Phelps et al (2017), Caperaa et al. (2025) and Privat et al. (2019), suggest that important developmental transitions occur between 9 and 21 dpf, with potentially important switches after 10 dpf. Including additional time points in this range could make the analysis even more informative, especially if baseline sensorimotor responses diverge substantially during this developmental window.

Minor comments
• Figures 1 and 2 are difficult to read because of the small font sizes and panel labelling. Improving the readability of these figures would help the reader.
• Are there baseline locomotor deficits in the mutant that could account for some of the reported effects?
• Is there a difference in swimming patterns or behaviour between the beginning and the end of each session? For example, do the animals show signs of habituation, fatigue, or reduced responsiveness over time?

Reviewer #2

(Remarks to the Author)
This manuscript presents an elegant and technically sophisticated analysis of the sensorimotor decision-making behavior in larval zebrafish using an optomotor response paradigm driven by random-dot-motion cues. The authors combine drift-diffusion modeling (DDM) with a Bayesian-optimization-based inference pipeline to uncover latent parameters governing motion integration and action selection. Importantly, many of these internal decision-making variables are not directly accessible through experiments alone, making the computational framework a major strength of the work.
Using this approach, the authors identify how specific latent sensorimotor parameters are altered during development as

well as following disease-associated genetic perturbations. The authors show that the integrator leak parameter is altered both across development and in mutants, and that these changes are associated with corresponding behavioral alterations. These results provide valuable mechanistic insight into the algorithmic implementation of sensorimotor integration in both healthy and disease contexts.
Overall, the analyses are carefully executed, the manuscript is clearly written and easy to follow, and the limitations of the study are carefully discussed. I believe this work will be of interest to researchers studying decision-making, computational modeling, and neurodevelopmental disorders.

Major comment:

One point that would benefit from clarification is the relationship between the inferred latent parameters and the observed behaviors. In particular, it is unclear how the developmental changes in Diffusion, Leak, and Delay (Fig. 4d–f) explain the increased swim frequency and lower percentage of correct swims shown in Fig. 4b,c.

Similarly, for the scn1lab +/- mutants, it is not immediately obvious how the less negative leak parameter accounts for the slight reduction in correct swims together with the increased inter-swim intervals. Intuitively, a more negative leak would enhance evidence integration (through recurrent network) and promote more accurate decisions. It would therefore help if the authors could more explicitly connect the inferred parameter changes to the observed behavioral phenotypes, for example through a more detailed explanation.

Minor comments:

1. The manuscript uses two scn1lab alleles; however, it is unclear whether both represent loss-of-function mutations. The authors should explicitly describe how the gene is affected in each mutant allele and clarify this in the Methods section.

2. The paragraph in lines 397–417 contains substantial interpretation of the results. For example, statements such as “This result may indicate that animals also … their nervous system activity becomes more dynamic” (lines 398–401) and “This finding may reflect increasing … matures during early development” are more appropriate for the Discussion section. I suggest moving these interpretative statements out of the Results section.

Reviewer #3

(Remarks to the Author)
Computational modeling of psychophysical responses is an important tool in cognition science. It can help to build hypotheses and provides insights even in the absence of neurophysiological recordings. The zebrafish is increasingly used in studies on decision making due to its experimental accessibility and the less complex brain of this small, non-cortical vertebrate. The senior author has a lot of expertise in modeling evidence accumulation (especially in diffusion drift models as also used here), and the data acquisition, analysis and computational modeling presented in the manuscript is of high standard. The authors first develop an experimental paradigm, in which the performance of individual animals (instead of population responses) can be recorded and then introduce a drift diffusion model that can very precisely extract relevant parameters that underlie the cognitive decision process. Only five parameters are needed to explain the observed turning behavior of animals during random dot visual stimuli: drift, noise, leak, delay, reset. The modeling shows a remarkable precision (small level of errors) as demonstrated in suitable simulations of the model. The authors then proceed to characterize the ontogeny of decision making during the optomotor response and furthermore use mutants to illustrate the usefulness of the approach to infer latent variables in cognitively impaired brains (e.g. in an epilepsy mutant). The authors report that older animals show altered algorithms for decision making: While the sensory representation of the motion stimuli tends to stay the same (drift parameter), swimming behavior is less related to the task in older animals (noise/diffusion parameter), and older animals appear to keep a short-term memory of accumulated evidence (negative leakage, positive feedback). Both changes (diffusion, leakage) can nicely be explained by changes in behavior (more complex behavior with additional tasks at later stages) and possible changes in the circuit (more recurrent connectivity or persistent activity at later stages, “priming”). The presented modeling approach is very useful and versatile for extracting these algorithmic differences. Of note, this study focused on behavioral responses and lays the ground for future neurophysiological studies. In conclusion, the approach is very powerful for building hypotheses regarding changing circuit algorithms, which can then nicely be proven or disproven via whole-brain imaging (as the authors’ lab has done in the past).

The study is well executed and the methodology is explained appropriately. The results will be of high interest to scientists interested in decision making and zebrafish circuits.

I also noticed one potential caveat that should be addressed in order to be able to uphold the claim regarding “priming”.

Major caveats:

1) Fig. 4b shows that the percentage of correct swims doesn’t change very much from 6 dpf to 9 dpf, so behavioral performance seems to be overall similar. Trial structure (Fig 1e) was such that many (thirty) swim events occurred during one motion stimulus phase, and the motion coherence and direction (relative to the animal) was kept constant during each motion stimulus phase. Given these facts, how well can the model distinguish between negative leakage (as seen for older animals) and larger drifts/smaller diffusion (as seen for younger animals)? Negative leakage and smaller drifts/larger diffusion will lead to opposing results: negative leakage (or larger drifts) will cause an earlier swim event. So, could the similar behavioral performance (Fig. 4b) be explained by negative leakage making up for smaller drifts or by negative

leakage making up for larger diffusion? This could be tested in the model in silico test runs (Figure 2) by checking for correlation of estimation errors of leakage and drift or anti-correlation of leakage and diffusion. [In Fig. 3f it looks like the parameters are correlated across fish, e.g. fish with higher diffusion coefficient also tend to have a higher drift coefficient.] Since most of the consecutive swims were executed during the same (continuous) motion direction, the leak parameter might be somewhat redundant with the drift or diffusion parameters. In the differential equation leakage is not dependent on the input (coherence level), while drift is, which would – in theory – argue against redundancy. But since the ISI and percentage correct swimming distributions are pretty flat for 50% and 100% coherence levels in older animals (Fig. 4b-c), the input (I) dependence might not play out as much as one would hope. In fact, since the mapping of the input strength is fixed, c.f. line 766, it seems plausible to the reviewer that the curve saturation in Fig. 4b for older animals has to be addressed by the model by introducing negative leaks, since the leak parameter does not depend on the input. If this caveat is substantiated by further analysis, the claimed “priming” effect in older animals might not exist. Another way to test for this caveat is to use saturating curves (for correct swimming as in Fig. 4b/8dpf) and use them in silico (instead of the Weber-Fechner law abiding “optimal” curves in Figure 2d) and then see whether this in silico simulation results in negative leaks. Or simply plot the % correct curves for all of the 100 simulated parameter sets and see whether positive leakage is possible for saturating curves.

Minor points (in chronological order):

Abstract: typical limited -- > typically limited?

In the intro, the reviewer suggest to state examples for animal behavior that are not temporally integrated in order to sharpen the readers understanding of what we are looking at in the manuscript and for what tasks integration is necessary.

l. 58f.: “map directly onto biological mechanisms”: if this algorithm is present in the brain, what would be an alternatively possible “mechanism”? Do you mean a different (synonymous) algorithm that only phenomenologically resembles diffusion drift?

l.85: priming-like -- > Since “priming” is a word that is more frequently used in psychology, the reviewer suggests to use an alternative word that relates to the fact that the priming stimulus in the manuscript is the same stimulus as the one that evidence is accumulated for. E.g. “unstable integrator”, “positive-feedback integrator”.

l.99: The anterior rhombencephalic turning region seems to oscillate spontaneously to drive spontaneous swimming, so for some swimming events, sensory accumulation might not be needed.

How about behavioral states as observed in Krishan et al. 2025? An animal can change its inter-swim interval slowly across time. The observed differences across animals in this study might be related to behavioral states instead (so the animals might be more similar overall than the parameter distributions suggest). This possibility should be mentioned.

Style: l.205…: “We then…We then”.

Figure 2: panel a is invading panel b, please amend.

l.329: what circuit mechanisms are meant here that could be revealed?

Fig 4e-f: are there interaction effects?

l.400 adult zebrafish are known to sometimes respond to OMR stimuli in the opposite direction (than expected). E.g. see https://pmc.ncbi.nlm.nih.gov/articles/PMC10962262/

l.: 843: for the modeling to work, the locomotor activity had to scale with coherence levels. While the fish in Fig. 3d did this, did the bulk of the fish (Fig.4) behave the same? The distributions for the population (analyzed in Fig. 4) should be shown as well.

l.434: what is “precise neurodevelopment”? Please rephrase.

Fig. 5b: Were scn1lab +/- mutants (allele 1) performing better (% correct swims) than wildtype siblings?

L.710: the cited article #6 doesn’t provide info on the stimulus. The following reference seems to be more suitable: Newsome, W. T. & Paré, E. B. J. Neurosci. 8, 2201–2211 (1988).

Extended Data Fig. 2a: Why were there some simulations that had negative drifts and positive leaks at the same time? These simulations should not have resulted in correct behavioral responses on average, and therefore should have been excluded from the simulation, shouldn’t they?

Please provide legends for the supplementary movies.

Version 1:

Reviewer comments:

Reviewer #2

(Remarks to the Author)
The authors have adequately addressed most of my comments.
I appreciate that the authors have provided more information on the behavioral phenotypes of the scn1lab mutants. However, the authors have not fully addressed my comment regarding whether these two mutants result in a loss or gain of function of the scn1lab gene. If this is not known, at least the authors should provide information on where the mutations were introduced using the CRISPR/Cas9 technique. In addition, for the allele generated by ZIRC (i.e., scn1laballele2), please provide the allele number that would allow readers to uniquely identify the same mutant used in this study. This information would be important for improving the reproducibility of the results presented in this manuscript.

Reviewer #3

(Remarks to the Author)
The authors have successfully addressed my major concern. The new supplementary figures now make it transparent to the readers which parameters are intrinsically correlated and which ones are not. Since the simulated data (Suppl. Fig 7) shows an almost saturated curve as well (percentage of correct trials at different coherence level) and negative and positive leak parameters are possible for such saturating curves, I am now convinced that the reported findings are justified and that the zebrafish behavior and corresponding parameters can be characterized well with the model.

Open Access This Peer Review File is licensed under a Creative Commons Attribution 4.0 International License, which permits use, sharing, adaptation, distribution and reproduction in any medium or format, as long as you give appropriate credit to the original author(s) and the source, provide a link to the Creative Commons license, and indicate if changes were made.
In cases where reviewers are anonymous, credit should be given to 'Anonymous Referee' and the source.
The images or other third party material in this Peer Review File are included in the article's Creative Commons license, unless indicated otherwise in a credit line to the material. If material is not included in the article's Creative Commons license and your intended use is not permitted by statutory regulation or exceeds the permitted use, you will need to obtain permission directly from the copyright holder.
To view a copy of this license, visit https://creativecommons.org/licenses/by/4.0/

** Please ensure you delete the link to your author homepage in this email if you wish to forward it to your coauthors **

Dear Professor Bahl,

Your manuscript entitled "Developmental and genetic modulation of evidence integration dynamics in zebrafish sensorimotor decision-making" has now been seen by 3 referees, whose comments are appended below. You will see from their comments below that while they find your work of considerable interest, some important points are raised. We are interested in the possibility of publishing your study in Communications Biology, but would like to consider your response to these concerns in the form of a revised manuscript before we make a final decision on publication.

We therefore invite you to revise and resubmit your manuscript, taking into account the points raised. In particular, please address all of the reviewers' concerns.

Please highlight all changes in the manuscript text file.

We are committed to providing a fair and constructive peer-review process. Do not hesitate to contact us if you wish to discuss the revision in more detail or if there are specific requests from the reviewers that you believe are technically impossible or unlikely to yield a meaningful outcome.

At the same time, we ask that you ensure your manuscript complies with our editorial policies. Specifically:

For all graphs depicting a single point value (e.g., mean) with error bars, you must add individual data points or convert the graph to a boxplot or dot-plot to show data distribution.

We report individual animal behavior in Fig. 1g. In later figures related to parameter extraction, we perform analyses on individual fish and report extracted values for individuals (Figs. 3–5). The reporting of the percentage correct and interbout interval as a function of coherence levels (Fig. 4b,c, Fig. 5b,c, Supplementary Fig. 3c,d, Supplementary Fig. 6) shows mean +/- std across fish. As we report individual animal parameters related to each animal, showing individual fish in these plots would unnecessarily clutter the display, and we would like to keep the current configuration.

It's mandatory to provide access to the numerical source data for graphs and charts either through a repository or by providing the data in a Supplementary Data file (in excel format).

We provide all numerical source data and source code to generate graphs, as described in our availability statement.

All blots/gels must be accompanied by size markers in every figure panel. Uncropped and unedited blot/gel images must be included as Supplementary Figure(s) in the Supplementary Information pdf.

Does not apply.

Please ensure that you have complied with the data deposition policies at the Nature Portfolio, please see here.

We provide access links to our data and source code for review. The DOI is already reserved and will become public upon publication.

Please ensure that you have complied with our policies on research involving animals and humans, see here

We detail the animal permits in the methods section.

Please follow the ARRIVE guidelines for reporting animal experiments. Please fully complete an ARRIVE checklist including both the essential and recommended set of items (adding information to the manuscript where needed) and upload this with your revised manuscript.

We have reviewed and completed the ARRIVE checklist and uploaded this with the revised manuscript.

Please also see our revision checklist for guidance on formatting the manuscript and complying with our policies. A comprehensive guide to our formatting requirements for final submissions is also available for your reference here.

We downloaded the revision checklist, and we comply with all editorial formatting requirements. The checklist is also uploaded.

Please use the following link to submit your revised manuscript, point-by-point response to the referees' comments (which should be in a separate document to the cover letter) and any additional files:

We have uploaded the revised manuscript, a new cover letter, and a point-by-point response to the referees' comments.

https://mts-commsbio.nature.com/cgi-bin/main.plex?el=A2Cx4SWM4A1BFIY4I7A9ftdXSKwhrgKV9LdbaYtKgJ82QZ

** This url links to your confidential home page and associated information about manuscripts you may have submitted or be reviewing for us. If you wish to forward this email to co-authors, please delete the link to your homepage first **

When submitting the revised version of your manuscript, please pay close attention to our Digital Image Integrity Guidelines.

We would like to receive your revision within 2 months, but appreciate that every situation is unique. We look forward to receiving your revised manuscript when it is ready, and will not enforce a hard deadline on this revision.

Please do not hesitate to contact me if you have any questions or would like to discuss these revisions further. We look forward to seeing the revised manuscript and thank you for the opportunity to review your work.

Best regards,

Jasmine Pan, PhD
Associate Editor
Communications Biology
orcid.org/0000-0002-7369-9225

Reviewers' comments:

Reviewer #1 (Remarks to the Author):

In this manuscript, Garza et al. apply a drift-diffusion framework to early larval sensorimotor responses. Their model extracts individual-level information from freely swimming larvae and further examines how these parameters change across development and in disease-associated mutants.

This is a very interesting manuscript that is likely to be of broad interest to both the zebrafish and neuroscience communities. The study is conceptually strong, and the approach is innovative. A few modifications could further strengthen the manuscript. They provide a solid framework with sound methotology and statistical analysis.

Major comments
• Given the broad scope of the journal, additional background on some of the core concepts would be helpful. In particular, the section discussing Bayesian optimization could be expanded to clarify why this approach was selected over alternative methods. Similarly, the Introduction may be easier to follow if the section beginning around line 55 were moved before line 46. Drift-diffusion is first introduced on line 46, but the manuscript does not yet provide sufficient context for non-expert readers on what this framework is and why it is useful in this setting.

We expanded the discussion related to Bayesian optimization (lines 178 and 770) and improved the structure of our introduction, as suggested by the referee (line 50).

• The developmental experiment tracking behaviour across time is interesting. However, other studies, including Roberts et al (2017), Cleal et al (2023), Phelps et al (2017), Caperaa et al. (2025) and Privat et al. (2019), suggest that important developmental transitions occur between 9 and 21 dpf, with potentially important switches after 10 dpf. Including additional time points in this range could make the analysis even more informative, especially if baseline sensorimotor responses diverge substantially during this developmental window.

We agree that it would be very informative to extend our experiments, analyses, and modeling across a much broader age range. In other projects in the lab, we are currently measuring behavior in zebrafish older than 14 dpf. These experiments indicate that the algorithms describing zebrafish behavior not only change gradually over time, but that they may be fundamentally replaced by new strategies. See Capelle et al. 2026 (*iScience*) for phototaxis. Similarly, we observe that the optomotor response becomes largely inverted in older fish, with regular internal-state-driven transitions, suggesting that the algorithms underlying motion-following may structurally change during development. We think that analysing these transitions within this current paper would lead to a much more complex story, and we would like to address these questions in another upcoming manuscript. We have now added a new discussion point (line 538) about the future extension of our age analyses and use this opportunity to also cite the recommended papers by the referee.

Minor comments
• Figures 1 and 2 are difficult to read because of the small font sizes and panel labelling. Improving the readability of these figures would help the reader.

Font sizes are standard 6pt. We have now increased the size of Figures 1 and 2, scaling all non-text graphical elements by 120%. We also moved all figures to the end of the manuscript to facilitate review.

• Are there baseline locomotor deficits in the mutant that could account for some of the reported effects?

We have quantified the baseline swim rates at 0 % coherence. In the mutant, inter-bout intervals (inverse of swim rate) are slightly increased (Fig. 5c). Our modeling suggests that these differences originate from internal parameter differences between control and mutants, which lead to less frequent threshold crossing in the mutant, also under non-motion conditions. No specific locomotor deficits beyond swim rate modulation (such as Tang et al, 2020, iScience) have been reported in the literature.

• Is there a difference in swimming patterns or behaviour between the beginning and the end of each session? For example, do the animals show signs of habituation, fatigue, or reduced responsiveness over time?

We have now quantified the inter-swim intervals for the first and second hour of the experiment in Supplementary Fig. 1a,b. We find that behavioral features like percentage of correct swims and inter-swim interval across coherence are preserved during the course of the experiment (line 128).

Reviewer #2 (Remarks to the Author):

This manuscript presents an elegant and technically sophisticated analysis of the sensorimotor decision-making behavior in larval zebrafish using an optomotor response paradigm driven by random-dot-motion cues. The authors combine drift-diffusion modeling (DDM) with a Bayesian-optimization-based inference pipeline to uncover latent parameters governing motion integration and action selection. Importantly, many of these internal decision-making variables are not directly accessible through experiments alone, making the computational framework a major strength of the work.
Using this approach, the authors identify how specific latent sensorimotor parameters are altered during development as well as following disease-associated genetic perturbations. The authors show that the integrator leak parameter is altered both across development and in mutants, and that these changes are associated with corresponding behavioral alterations. These results provide valuable mechanistic insight into the algorithmic implementation of sensorimotor integration in both healthy and disease contexts.
Overall, the analyses are carefully executed, the manuscript is clearly written and easy to follow, and the limitations of the study are carefully discussed. I believe this work will be of interest to researchers studying decision-making, computational modeling, and neurodevelopmental disorders.

Major comment:

One point that would benefit from clarification is the relationship between the inferred latent parameters and the observed behaviors. In particular, it is unclear how the developmental changes in Diffusion, Leak, and Delay (Fig. 4d–f) explain the increased swim frequency and lower percentage of correct swims shown in Fig. 4b,c.

We now provide a more intuitive description of the relationship between model parameters and observable behavior, allowing readers to appreciate better how developmental changes affect behavior (line 276). We acknowledge that linking model parameters to behavioral outcomes is important, and we now provide a new stand-alone graphical user interface to explore such relationships in real-time (lines 158 and 734). This software will help readers to validate our descriptions related to the age analyses and other aspects of the manuscript. We also think that providing such a framework will facilitate teaching on drift-diffusion models in the academic environment.

Similarly, for the scn1lab +/- mutants, it is not immediately obvious how the less negative leak parameter accounts for the slight reduction in correct swims together with the increased inter-swim intervals. Intuitively, a more negative leak would enhance evidence integration (through recurrent network) and promote more accurate decisions. It would therefore help if the authors could more explicitly connect the inferred parameter changes to the observed behavioral phenotypes, for example through a more detailed explanation.

We apologize for not being clear enough. We now state that changes are represented by larger inter-swim intervals, with a minor increase in the percentage of correct swims (line 395). A less negative leak parameter would lead to less frequent threshold crossing as the self-reinforcing property of the integrator variable would be reduced. As such, these fish would swim less often and be more accurate. We have mentioned these points in the updated results section and discussion (line 390 and 520). Generally, a more negative leak makes the animal more impulsive, and thus it can amplify mistakes. The graphical user interface we now provide should further help the reader's understanding, allowing them to first-hand simulate and visualize the model in such configurations.

Minor comments:

1. The manuscript uses two scn1lab alleles; however, it is unclear whether both represent loss-of-function mutations. The authors should explicitly describe how the gene is affected in each mutant allele and clarify this in the Methods section.

We now address the behavioral impact of both these mutations (line 682). In the Methods section, we refer to the origin of the two lines (line 680).

2. The paragraph in lines 397–417 contains substantial interpretation of the results. For example, statements such as "This result may indicate that animals also … their nervous system activity becomes more dynamic" (lines 398–401) and "This finding may reflect increasing … matures during early development" are more appropriate for the Discussion section. I suggest moving these interpretative statements out of the Results section.

As suggested, we moved the mentioned interpretative sentences to the Discussion section (lines 532 to 544).

Reviewer #3 (Remarks to the Author):

Computational modeling of psychophysical responses is an important tool in cognition science. It can help to build hypotheses and provides insights even in the absence of neurophysiological recordings. The zebrafish is increasingly used in studies on decision making due to its experimental accessibility and the less complex brain of this small, non-cortical vertebrate. The senior author has a lot of expertise in modeling evidence accumulation (especially in diffusion drift models as also used here), and the data acquisition, analysis and computational modeling presented in the manuscript is of high standard. The authors first develop an experimental paradigm, in which the performance of individual animals (instead of population responses) can be recorded and then introduce a drift diffusion model that can very precisely extract relevant parameters that underlie the cognitive decision process. Only five parameters are needed to explain the observed turning behavior of animals during random dot visual stimuli: drift, noise, leak, delay, reset. The modeling shows a remarkable precision (small level of errors) as demonstrated in suitable simulations of the model. The authors then proceed to characterize the ontogeny of decision making during the optomotor response and furthermore use mutants to illustrate the usefulness of the approach to infer latent variables in cognitively impaired brains (e.g. in an epilepsy mutant). The authors report that older animals show altered algorithms for decision making: While the sensory representation of the motion stimuli tends to stay the same (drift parameter), swimming behavior is less related to the task

in older animals (noise/diffusion parameter), and older animals appear to keep a short-term memory of accumulated evidence (negative leakage, positive feedback). Both changes (diffusion, leakage) can nicely be explained by changes in behavior (more complex behavior with additional tasks at later stages) and possible changes in the circuit (more recurrent connectivity or persistent activity at later stages, “priming”). The presented modeling approach is very useful and versatile for extracting these algorithmic differences. Of note, this study focused on behavioral responses and lays the ground for future neurophysiological studies. In conclusion, the approach is very powerful for building hypotheses regarding changing circuit algorithms, which can then nicely be proven or disproven via whole-brain imaging (as the authors’ lab has done in the past).

The study is well executed and the methodology is explained appropriately. The results will be of high interest to scientists interested in decision making and zebrafish circuits.

I also noticed one potential caveat that should be addressed in order to be able to uphold the claim regarding “priming”.

Major caveats:

1) Fig. 4b shows that the percentage of correct swims doesn’t change very much from 6 dpf to 9 dpf, so behavioral performance seems to be overall similar. Trial structure (Fig 1e) was such that many (thirty) swim events occurred during one motion stimulus phase, and the motion coherence and direction (relative to the animal) was kept constant during each motion stimulus phase. Given these facts, how well can the model distinguish between negative leakage (as seen for older animals) and larger drifts/smaller diffusion (as seen for younger animals)? Negative leakage and smaller drifts/larger diffusion will lead to opposing results: negative leakage (or larger drifts) will cause an earlier swim event. So, could the similar behavioral performance (Fig. 4b) be explained by negative leakage making up for smaller drifts or by negative leakage making up for larger diffusion? This could be tested in the model in silico test runs (Figure 2) by checking for correlation of estimation errors of leakage and drift or anti-correlation of leakage and diffusion. [In Fig. 3f it looks like the parameters are correlated across fish, e.g. fish with higher diffusion coefficient also tend to have a higher drift coefficient.] Since most of the consecutive swims were executed during the same (continuous) motion direction, the leak parameter might be somewhat redundant with the drift or diffusion parameters. In the differential equation leakage is not dependent on the input (coherence level), while drift is, which would – in theory – argue against redundancy. But since the ISI and percentage correct swimming distributions are pretty flat for 50% and 100% coherence levels in older animals (Fig. 4b-c), the input (I) dependence might not play out as much as one would hope. In fact, since the mapping of the input strength is fixed, c.f. line 766, it seems plausible to the reviewer that the curve saturation in Fig. 4b for older animals has to be addressed by the model by introducing negative leaks, since the leak parameter does not depend on the input. If this caveat is substantiated by further analysis, the claimed “priming” effect in older animals might not exist. Another way to test for this caveat is to use saturating curves (for correct swimming as in Fig. 4b/8dpf) and use them in silico (instead of the Weber-Fechner law abiding “optimal” curves in Figure 2d) and then see whether this in silico simulation results in negative leaks. Or simply plot the % correct curves for all of the 100 simulated parameter sets and see whether positive leakage is possible for saturating curves.

While both a more negative leak and a larger drift increase decision frequency, they play distinct roles: drift selectively increases the proportion of correct swim events, amplifying their inter-swim interval distributions while suppressing those associated with incorrect events, whereas leak magnitude modulates overall swim frequency symmetrically, without biasing event sign. In agreement with this, the correlation analysis of synthetic datasets added in the Appendix (Supplementary Fig. 8b) shows no significant correlation between leak-drift or leak-diffusion pairs. The near-optimal identifiability shown in Supplementary Fig. 4c,d reveals minor redundancies among parameters, but we deliberately refrained from further model reduction, as collapsing parameters would reduce their individual interpretability. To help readers develop an intuition for each parameter's contribution, we now provide an interactive graphical user interface for simulating the model under custom parameterizations and stimulus conditions (Supplementary Fig. 2). Furthermore, we now publish Supplementary Data 1 reporting target and estimated parameters for all the synthetic models shown in Supplementary Fig. 4. Finally, we drafted Supplementary Fig. 7 showing the existence of saturating psychometric curves for both positive and negative leak (lines 335 to 340).

Minor points (in chronological order):

Abstract: typical limited -- > typically limited?

Corrected.

In the intro, the reviewer suggest to state examples for animal behavior that are not temporally integrated in order to sharpen the readers understanding of what we are looking at in the manuscript and for what tasks integration is necessary.

We now refer to non-integrating behavior in line 34.

l. 58f.: “map directly onto biological mechanisms”: if this algorithm is present in the brain, what would be an alternatively possible “mechanism”? Do you mean a different (synonymous) algorithm that only phenomenologically resembles diffusion drift?

We toned down such statements in our updated introduction and now clarify these points at line 62.

l.85: priming-like -- > Since “priming” is a word that is more frequently used in psychology, the reviewer suggests to use an alternative word that relates to the fact that the priming stimulus in the manuscript is the same stimulus as the one that evidence is accumulated for. E.g. “unstable integrator”, “positive-feedback integrator”.

We agree with the referees’ assessment and have adjusted our wording to positive-feedback integrator throughout the manuscript. The only place where we now hint at potential relations to priming effects is in our updated discussion section (line 525).

l.99: The anterior rhombencephalic turning region seems to oscillate spontaneously to drive spontaneous swimming, so for some swimming events, sensory accumulation might not be needed.

We thank the reviewer for this important clarification. In fact, not all swimming events in larval zebrafish may be sensory-driven. The anterior rhombencephalic turning region (ARTR) oscillates spontaneously and can drive turning behavior and swimming bouts in the absence of sensory input (Dunn et al., 2016; Wolf et al., 2017). This means that a subset of swimming events in our data may reflect spontaneous motor output rather than the outcome of sensory evidence accumulation. We now acknowledge this explicitly (line 103) and note that our drift-diffusion framework is intended to describe the sensory-driven component of the decision process. The stochastic term in the DDM can, in part, absorb spontaneous, internally driven swim initiations as noise. Indeed, the baseline drift and diffusion parameters may reflect a mixture of sensory accumulation and intrinsic motor variability. Disentangling these two sources rigorously would require simultaneous neural recordings, which is beyond the scope of the current study. We have added a note in the Discussion (line 565) acknowledging that spontaneous ARTR-driven swimming events may contribute to the behavioral variability we observe, and that future work combining neural imaging with behavioral modeling will be needed to separate sensory-driven from internally generated decisions.

How about behavioral states as observed in Krishan et al. 2025? An animal can change its inter-swim interval slowly across time. The observed differences across animals in this study might be related to behavioral states instead (so the animals might be more similar overall than the parameter distributions suggest). This possibility should be mentioned.

We agree with the referee that behavioral states may influence the interpretation of the inferred parameters and explain some aspects of the observed inter-individual differences. We now mention this point in our updated discussion (line 494).

Style: l.205…: “We then…We then”.

Corrected.

Figure 2: panel a is invading panel b, please amend.

Corrected.

l.329: what circuit mechanisms are meant here that could be revealed?

We now provide examples at line 276.

Fig 4e-f: are there interaction effects?

We have now performed careful new analyses of the interaction effects. Indeed, we identify some biologically relevant parameter co-variations that cannot be explained by model design and potential fitting artifacts. However, the description of these points requires major explanations. Thus, to preserve the readability of the main paper, we have moved this new section into a new Appendix part, within our updated supplement. We refer to the question on parameter interactions in the paragraph related to the age analysis (line 341).

l.400 adult zebrafish are known to sometimes respond to OMR stimuli in the opposite direction (than expected). E.g. see https://pmc.ncbi.nlm.nih.gov/articles/PMC10962262/

We now acknowledge this phenomenon at line 538.

l.: 843: for the modeling to work, the locomotor activity had to scale with coherence levels. While the fish in Fig. 3d did this, did the bulk of the fish (Fig.4) behave the same? The distributions for the population (analyzed in Fig. 4) should be shown as well.

We quantify activity and performance by computing fish-specific 'percentage correct' and 'inter-swim intervals', based on distributions as the example ones shown in Fig. 3d. The bulk summary for the population is shown in Fig. 4b,c. The percentage of correct swims increases, and the interbout interval (inverse of locomotor activity) decreases with coherence.

l.434: what is "precise neurodevelopment"? Please rephrase.

Corrected.

Fig. 5b: Were scn1lab +/- mutants (allele 1) performing better (% correct swims) than wildtype siblings?

Yes, this result is consistent with what was reported in Harpaz et al. 2021, the study originally acquiring and analyzing this dataset.

L.710: the cited article #6 doesn't provide info on the stimulus. The following reference seems to be more suitable: Newsome, W. T. & Paré, E. B. J. Neurosci. 8, 2201–2211 (1988).

Corrected.

Extended Data Fig. 2a: Why were there some simulations that had negative drifts and positive leaks at the same time? These simulations should not have resulted in correct behavioral responses on average, and therefore should have been excluded from the simulation, shouldn't they?

The criteria for selecting randomly generated parameter sets (Methods: Criteria for selecting target models) do not exclude negative drift values. As already discussed in a previous point, antifollowing behavior, corresponding to negative drift, has indeed been reported in zebrafish engaged with whole-field motion stimulation, and including such parameter regimes allows our framework to capture a wider portion of the behavioral repertoire observed experimentally.

Please provide legends for the supplementary movies.

We now provide legends in a document shared alongside the supplementary movies.

** Please ensure you delete the link to your author homepage in this email if you wish to forward it to your coauthors **

Dear Professor Bahl,

Thank you again for submitting your revised manuscript "Developmental and genetic modulation of evidence integration dynamics in zebrafish sensorimotor decision-making" to Communications Biology. We have now received reports from 2 of the previous reviewers. You will find the reviewers' reports below. As you can see, they are now recommending publication, pending addressing Reviewer #2's final minor recommendation for transparency and reproducibility.

We therefore invite you to address Reviewer #2's last requests and revise your paper to comply with editorial policies and formatting requirements and to maximise the accessibility and therefore the impact of your work.

Please edit your revised manuscript according to requests listed in the online table, https://springernature.sharepoint.com/:w:/s/NaturePortfolioERTSite/IQDi4bCXy96RTIdMyN1V2tHdAZMcJ2QSOBANbbcA7ovnPbw?e=29g45L>here.

Please **read and complete the table in the link**, as we will be unable to further assess your revised manuscript until these editorial requirements are addressed, and your manuscript will be returned to you for further action.

We have edited the revised manuscript according to these requests.

Please outline all revisions made in the right-hand column of the table and include any relevant notes that may aid us in assessing your revised work.

When resubmitting, please include:

 A point-by-point response to the reviewers' comments. If you are unable to address specific reviewer requests or find any points invalid, please explain
Is attached.

 A clean version of your revised manuscript with no mark-ups

Is attached.

 A marked-up version of your paper with all changes highlighted in a different colour
Is attached.

Please use the link below to submit your revised files:

https://mts-commsbio.nature.com/cgi-bin/main.plex?el=A7Cx6SWM7B3BFIY2I1A9ftdXSKwhrgKV9LdbaYtKgJ82QZ

** This url links to your confidential home page and associated information about manuscripts you may have submitted or that you are reviewing for us. If you wish to forward this email to co-authors, please delete the link to your homepage first **

We hope to receive your revised paper within six weeks, but we understand that the revisions may take longer. Please let us know if you find that the revision process will take substantially more time.

We are committed to providing a fair and constructive peer-review process. Please do not hesitate to contact me if you have any questions or would like to discuss these revisions further. We look forward to seeing the revised manuscript and thank you for the opportunity to review your work.

Best regards,

Jasmine Pan, PhD
Senior Editor
Communications Biology
orcid.org/0000-0002-7369-9225

Reviewers' comments:

Reviewer #2 (Remarks to the Author):

The authors have adequately addressed most of my comments.
I appreciate that the authors have provided more information on the behavioral phenotypes of the scn1lab mutants. However, the authors have not fully addressed my comment regarding whether these two mutants result in a loss or gain of function of the scn1lab gene. If this is not known, at least the authors should provide information on where the mutations were introduced using the CRISPR/Cas9 technique. In addition, for the allele generated by ZIRC (i.e., scn1laballele2), please provide the allele number that would allow readers to uniquely identify the same mutant used in this study. This information would be important for improving the reproducibility of the results presented in this manuscript.

We have now provided the required allele information for all transgenic lines, which are now listed in detail in the updated method section (lines 678 and following).

Reviewer #3 (Remarks to the Author):

The authors have successfully addressed my major concern. The new supplementary figures now make it transparent to the readers which parameters are intrinsically correlated and which ones are not. Since the simulated data (Suppl. Fig 7) shows an almost saturated curve as well (percentage of correct trials at different coherence level) and negative and positive leak parameters are possible for such saturating curves, I am now convinced that the reported findings are justified and that the zebrafish behavior and corresponding parameters can be characterized well with the model.
