# Supplementary material for: Developmental and genetic modulation of evidence integration dynamics in zebrafish sensorimotor decision-making
Source: Commun Biol. 2026 Oct 29;9:1435. doi: 10.1038/s42003-026-11264-y (PMC13723567; doi:10.1038/s42003-026-11264-y)
Supplement: Supplementary file 2 [file 42003_2026_11264_MOESM2_ESM.pdf]

SUPPLEMENTARY MATERIAL

Legends for Supplementary Movies 1-3 and Supplementary Data 1

Supplementary Figs. 1–8

Appendix

Developmental and genetic modulation of evidence integration dynamics in zebrafish sensorimotor decision-making

AUTHORS

Roberto Garza[1,2,4,*], Ahmed El Hady[2,4,‡], Armin Bahl[1,2,3,4,‡,*]

AFFILIATIONS

[1] Department of Biology, University of Konstanz, Germany

[2] Centre for the Advanced Study of Collective Behaviour, University of Konstanz, Germany

[3] Zukunftskolleg, University of Konstanz, Germany

[4] International Max Planck Research School for Quantitative Behaviour, Ecology and Evolution (IMPRS/QBEE); Max Planck Institute of Animal Behavior, Konstanz, Germany

[‡] These authors jointly supervised this work

* Correspondence to roberto.garza@uni-konstanz.de; armin.bahl@uni-konstanz.de

Supplementary Movie 1: Video recording of a freely swimming 5-day-old larval zebrafish on top of a random-dot-motion stimulus (coherence level 50%, rightward). At every frame, each dot gets redrawn, either with a small spatial offset or at a random new location in the arena, depending on the level of coherence. We display 10 s of 0% coherence, followed by 30 s of coherent motion (illustrated by red text appearing next to time), followed by 10 s of 0% coherence. Movement updates in real-time such that direction is always perpendicular to the body orientation of the animal, allowing controlled stimulus delivery across consecutive swim decision events. The video recording of the larval zebrafish (light brown-colored) and the random-dot-motion stimulus are overlaid, together with the animal's trajectory over the recent 10 s in blue. The left video shows the experimenter's view. The right videos are real-time renderings of the visual scene, approximating the visual perspective of the animal. The fields of view are illustrated by red-shaded arcs, where each eye covers one hemisphere with a small binocular overlap. The two eyes only see a limited part of the scene, from which animals extract the overall motion signal through temporal integration. Note that in these projections, leftward and rightward motion cues should be visible as receding or approaching dots.

Supplementary Movie 2: Same as Supplementary Movie 1, but for random-dot-motion at coherence level 25%, drifting leftward.

Supplementary Movie 3: Same as Supplementary Movie 1, but for random-dot-motion at coherence level 0%.

Supplementary Data 1: Table of randomly generated target parameter sets together with inferred values after optimization. Related to **Supplementary Fig. 4a,b**.

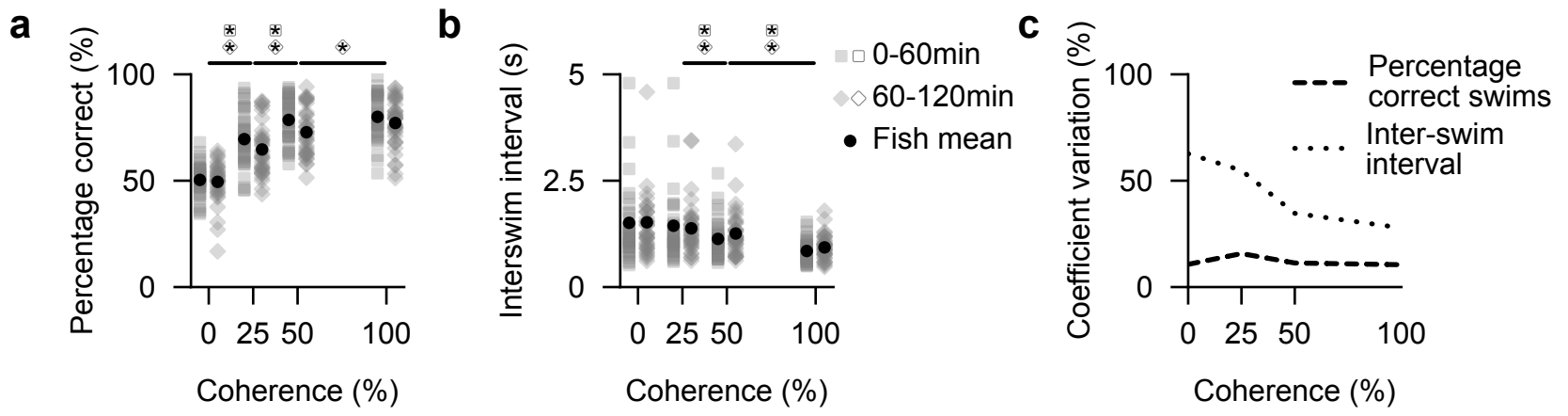

Supplementary Fig. 1 | Quantification of behavioral variability in experimental time and across fish. a, Percentage of correct swims as a function of coherence level, computed separately for the first (squares) and the second (diamonds) 60-minute intervals of the experiment. **b**, Same as in (a), but for inter-swim interval. **a,b**, For each coherence level and time bin, individual fish values are represented by gray markers; the associated population averages are shown as black circles. Distributions at adjacent coherence levels were compared using a two-sided Wilcoxon signed-rank test; asterisks indicate a significant difference between distributions ($p < 0.05$), enclosed in squares or diamonds to denote significance for each time interval. **c**, Coefficients of variation of percentage correct (dashed line) and inter-swim interval (dotted line) for different coherence levels. N = 39 larvae. Same animals as in related **Fig. 1g**.

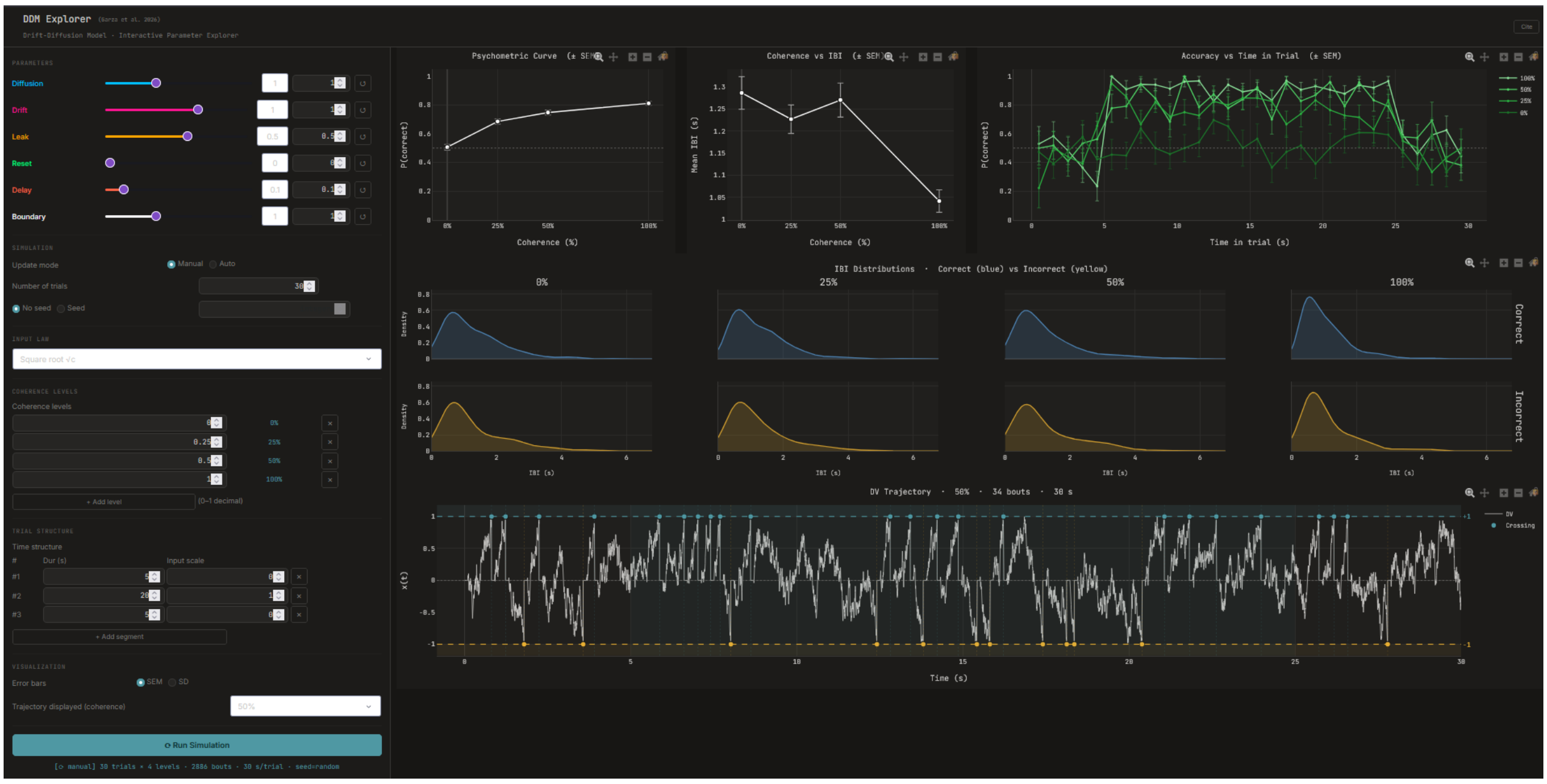

Supplementary Fig. 2 | Interactive browser-based graphical user interface for in silico experiments with drift-diffusion models. The Python-based software can be downloaded with detailed instructions from our persistent repository: https://doi.org/10.48606/rbfa7tt6aez1pjtj.

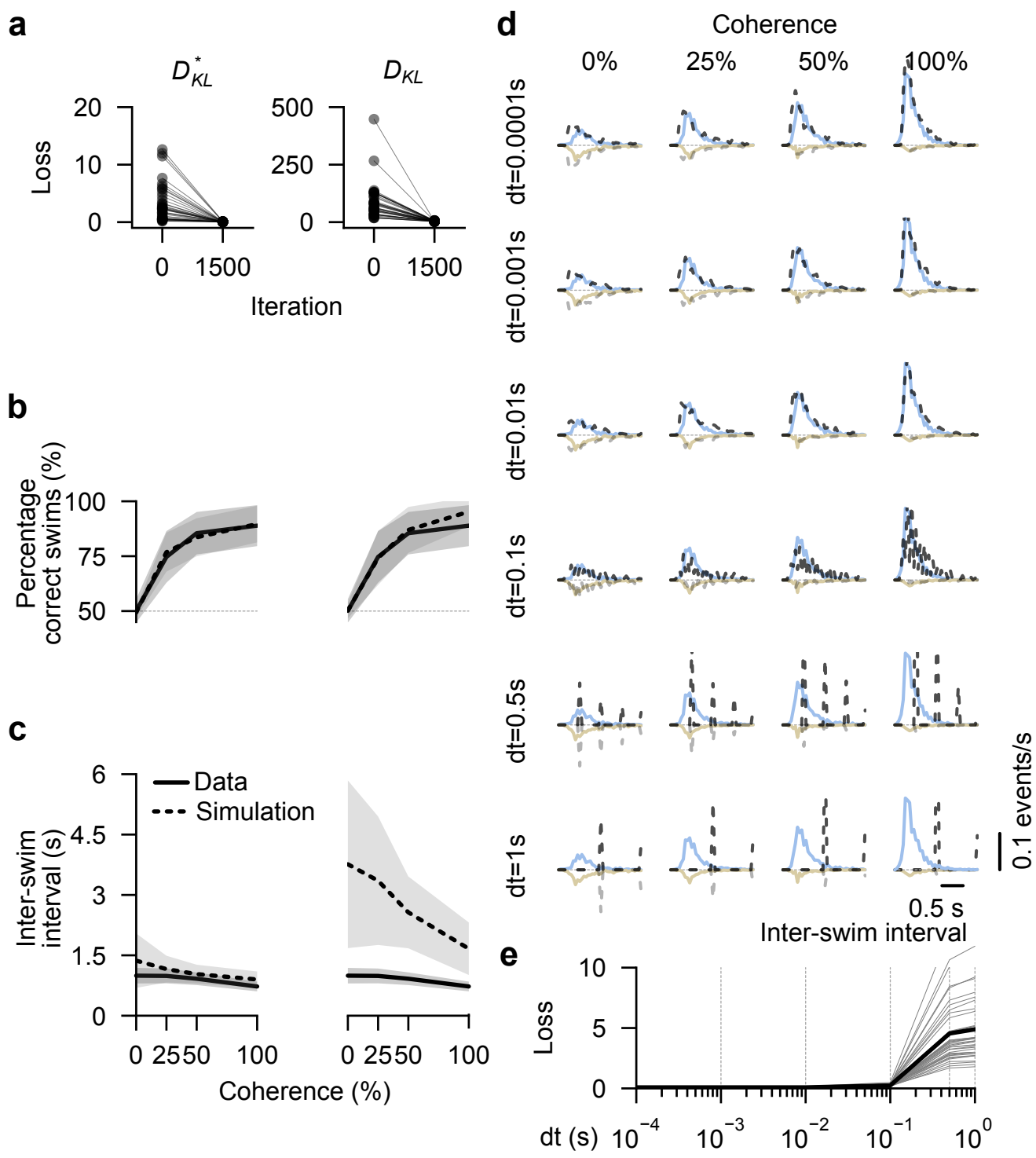

Supplementary Fig. 3 | Optimization results with two different loss functions and simulation time steps. a, Loss function reduction before and after fit of experimental data from N=39 5-dpf real larvae, using the improved (D^*_{KL}) and standard (D_{KL}) forms of the Kullback-Leibler divergence (**Methods**). Despite their difference in initial value, both loss functions converge to near-zero. **b,c**, Percentage of correct swims and inter-swim intervals plotted against the coherence level for the experimental (solid line) and fitted model (dashed line). The shaded areas surrounding each line represent the respective standard deviations across fish and fitted models. Optimization using D^*_{KL} leads to better matches of the target experimental data (left side) compared to results obtained using D_{KL} (right side). **d**, Model fits using different simulation time resolutions dt (labeled on the left side of each row). The target dataset was always the same experimental real fish. **e**, Final loss as a function of dt for all N=39 5-dpf real fish. Thin gray lines indicate single fish; the thick black line is their average. The final loss stably remains near zero for $dt \leq 0.01\ s$. All fish are the same individuals as in **Fig. 1g**. Related to **Figs. 1** and **2.**

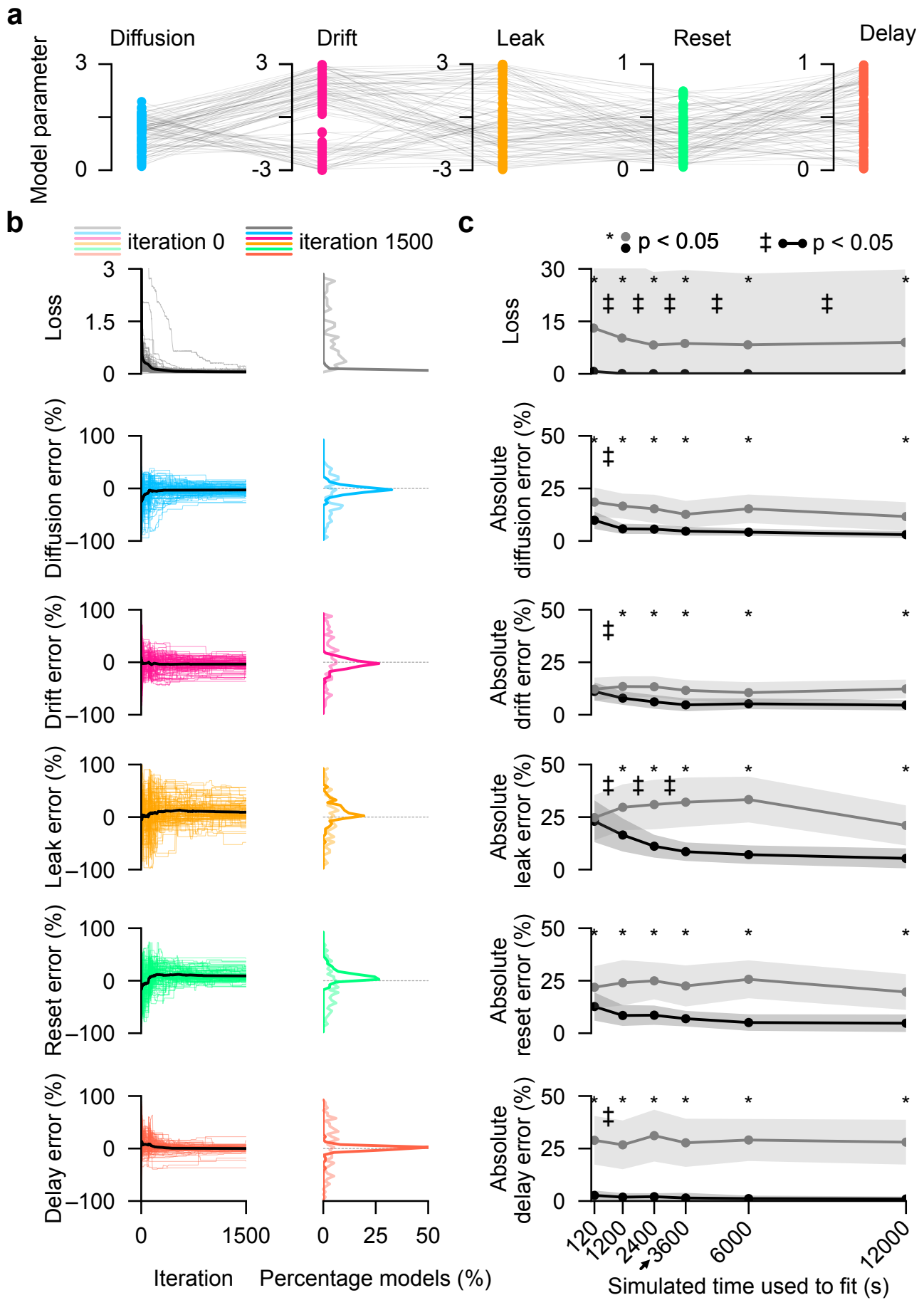

Supplementary Fig. 4 | Bayesian optimization can extract latent model parameters from a broad range of datasets using experimentally feasible time scales. a, Parameter sets of 100 randomly generated models. These models fulfill a list of criteria to make them biologically realistic but are not fit to experimental data (**Methods**). Gray lines connect values of the parameters belonging to the same model. **b,** Left: Evolution of loss and parameter estimation error over optimization iterations. Colored thin lines represent individual model fits. The black line shows the median across models. Right: Distribution of values at the beginning of (light colors) and after (dark colors) optimization. See **Supplementary Data 1** for tabular comparisons of target and fitted parameters. **c,** Loss and absolute parameter estimation errors obtained for datasets of different simulation lengths. For each length, we used the same 100 randomly generated models as in (a). The black line represents the average value across models. The black shaded area represents the corresponding standard deviation. As a control, in gray, the same results are shown for randomly initialized models before optimization. Asterisks (*) indicate significant differences ($p < 0.05$), comparing randomly initialized values to optimized ones. Double crosses (‡) display significant changes ($p < 0.05$), comparing optimized results from one simulation length to the next. Two-sided bootstrapping hypothesis testing was used in both cases. The arrow points to the size of experimental datasets. Related to **Fig. 2**.

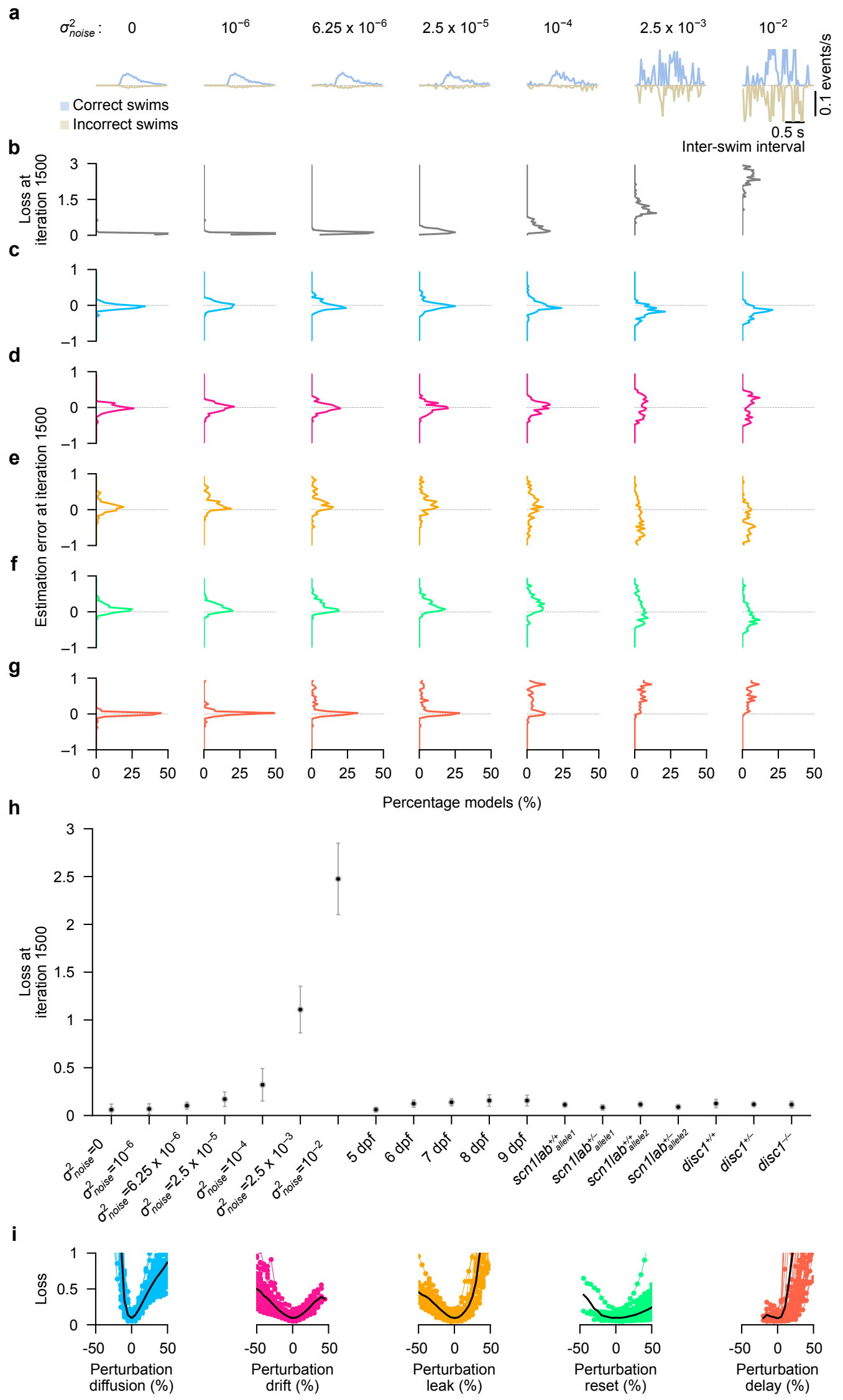

Supplementary Fig. 5 | Bayesian optimization is robust to noise and generalizes across experimental conditions. **a**, Distributions of inter-swim intervals for correct swims (blue) and incorrect swims (gold) at 50% coherence from an example simulated model. We added different amounts of Gaussian noise to the distributions. σ^2_{noise} indicates the noise variance. **b–g**, Distribution of loss function value and parameter estimation errors after optimization for each noise level for 100 randomly generated models. Same models as in **Supplementary Fig. 4a**. **h**, Average loss function value after optimization for simulations with added Gaussian noise, quantified from (b), as well as for various experimental conditions tested in the paper. Error bars represent standard deviation. **i,** Change of loss for small parameter perturbations after fitting models to experimental data from N=39 5 dpf real larvae (same individuals as in **Fig. 1g**). Perturbations are measured in percentage of the width of each parameter's search space. Thin colored lines link individual loss estimations (colored circles) of specific perturbation percentages for each fish and parameter. Related to **Figs. 2–5**.

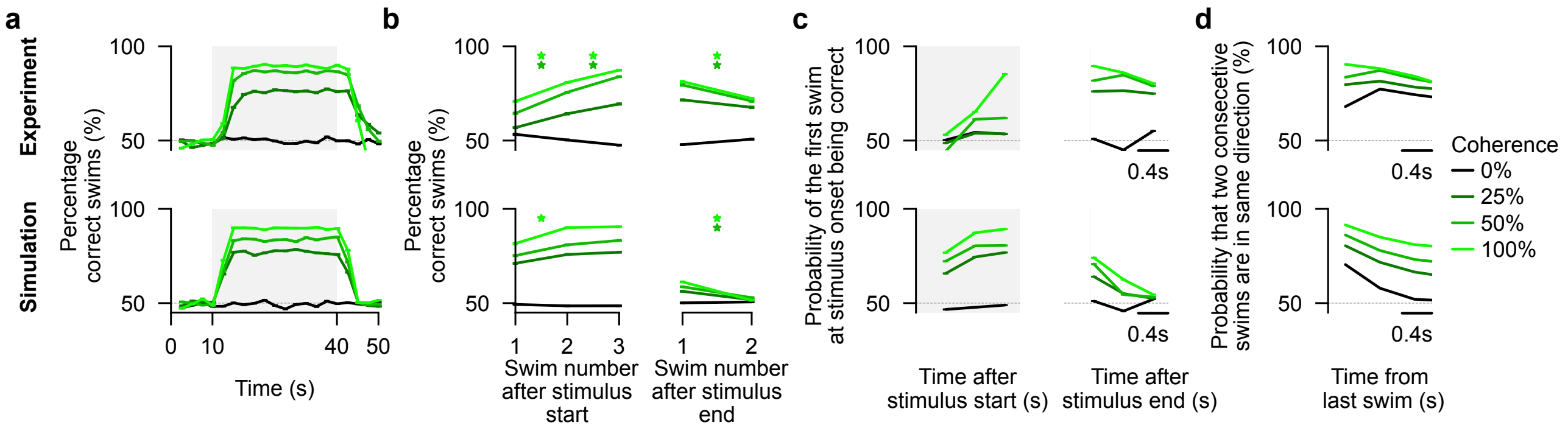

Supplementary Fig. 6 | Fitted drift-diffusion models can predict behavioral features not explicitly used during model generation. a, Percentage of correct swims, binned over time (with bins spanning 2.5 s) for all tested coherence levels. Gray boxes indicate the period of motion coherence display. Before and after, we display 0% coherence. The slow rise and decay of the curve at the start and stop of stimulus presentation indicate temporal integration and persistent motion memory. **b**, Percentage of correct swims increasing in the first three swims after the start of the stimulus (left) and decreasing in the first two swims after the end of the stimulus (right). Asterisks (*) indicate significant ($p < 0.05$, two-sided bootstrapping hypothesis test) changes in performance from one swimming event to the next at a given coherence level (different green levels). **c**, Probability of the first swim after the stimulus start (left) and after the stimulus end (right) to be correct. Swimming events were binned depending on the delay relative to the stimulus start (left) or end (right). **d,** Probability to consecutively swim in the same direction as a function of inter-swim interval during periods of 0% coherence. The slow decay indicates the tendency to consecutively repeat the same sensorimotor decision. The top row in (a–d) represents results from experimental data, merged from all animals. The bottom row in (a–d) shows model-generated results, merged from individual model simulations. N=39 fish and N=39 individual fish-fitted models, respectively. All experimental animals were 5 dpf old. Same animals and optimized models as in related **Fig. 3**.

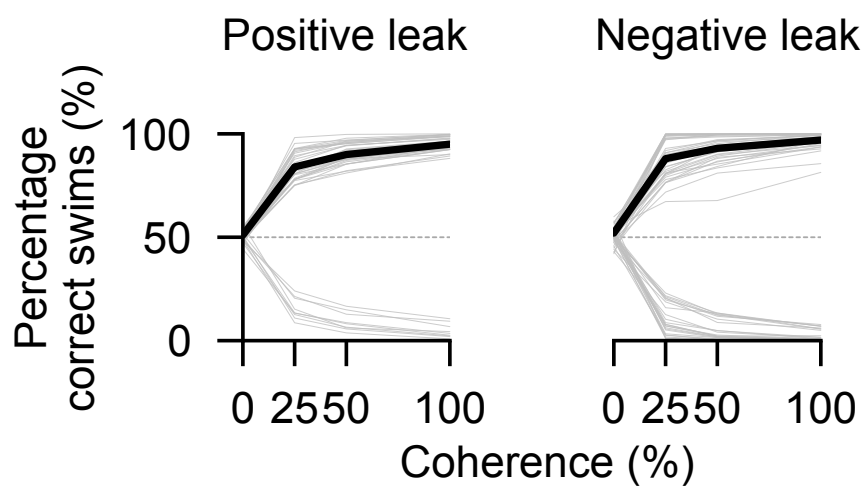

Supplementary Fig. 7 | Curve saturation does not entail negative leak values. Percentage of correct swims as a function of coherence level for synthetic models with positive (left) and negative (right) leak. Both parameter regimes yield saturating curves, demonstrating that saturation alone is insufficient to drive negative leak estimates. The model set is identical to that shown in **Supplementary Fig. 4a**. Gray lines indicate curves for individual models; the flipped population average (with all curves below 50% mirrored with respect to the 50% line) is shown in black. Models with a negative drift are allowed in the set, resulting in accuracy curves that decrease with increasing coherence.

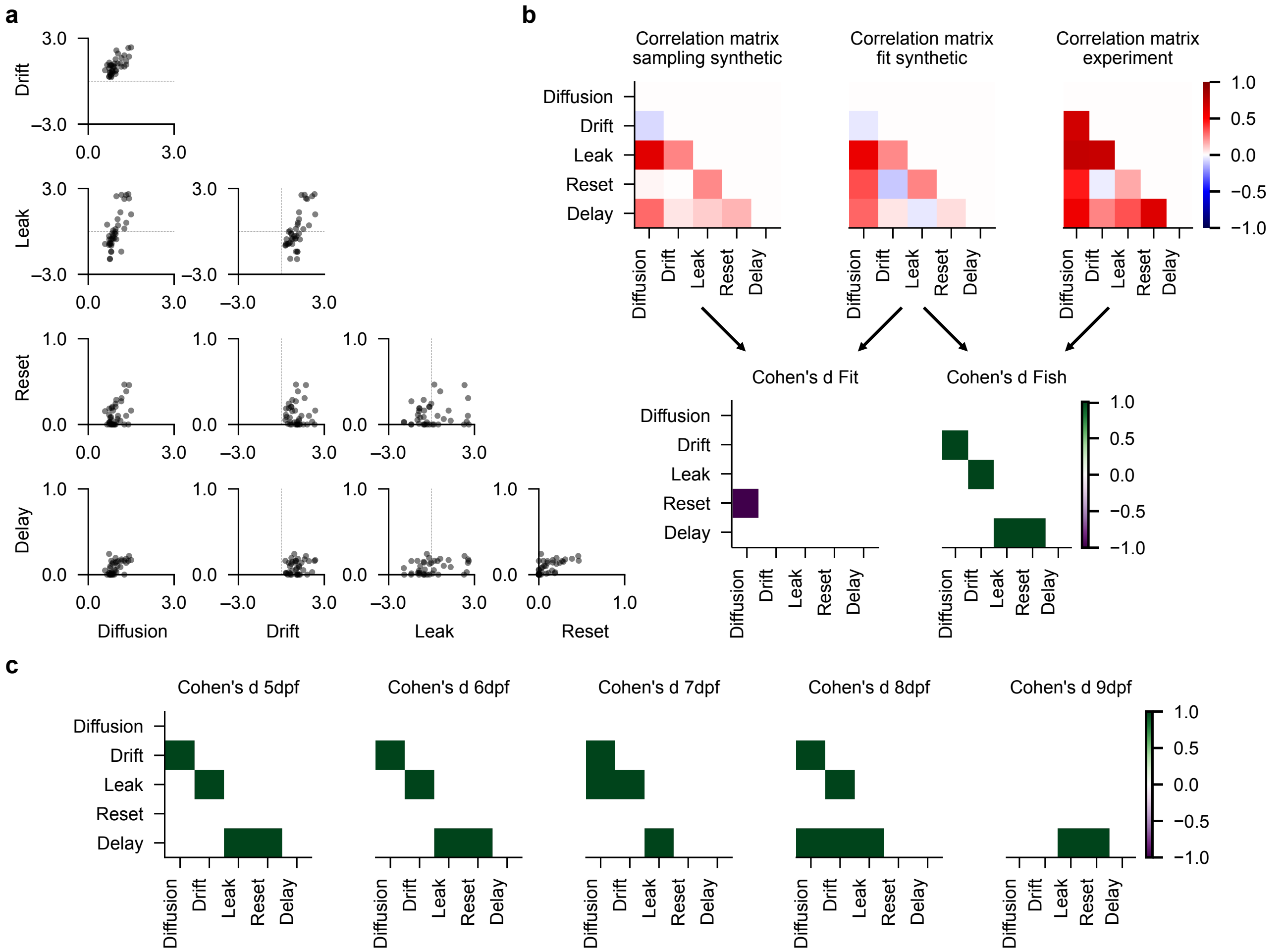

Supplementary Fig. 8 | Drift-diffusion model parameters extracted from behavioral data correlate more than expected by chance. a, Pairs of estimated parameters for N=39 5-dpf animals plotted against each other (same animals as in **Fig. 3f,g**). Correlation values can be seen in (b), top. **b**, Parameter correlation matrices (top), analyzing the effect of model selection criteria (top-left), fitting pipeline of model-generated data (top-middle), in comparison to the real experimental data (top-right). Using two-sided bootstrapping tests on repeated fitting trials (**Methods**), we identify correlations that are significantly different between matrices ($p < 0.01$). For significant values, we calculate Cohen's d as a measure of effect strength (bottom). Non-significant values are not shown. **c**, Significant parameter correlation effect strengths, comparing experimental conditions across all tested age groups and fitted model-generated data. Same analysis as in the bottom right matrix in (b). Same animals as in **Fig. 4**.

APPENDIX

Extracted parameter correlations are higher than expected by chance

Our drift-diffusion model uses five parameters to describe the dynamics of the sensorimotor decision-making process, matching experimental data across a wide range of conditions (**Figs. 4** and **5**). We next asked whether certain parameter configurations are biologically coupled within individual animals. For instance, larvae might compensate for a noisy integrator with stronger drift or compensate for stronger drift with more leaky dynamics. Such parameter dependencies could point to specific developmental programs or regulatory mechanisms that actively tune cognitive algorithms to environmental demands.

Computing correlations between pairs of estimated parameters in 5-dpf wild-type larvae revealed that most parameter pairs were positively correlated (**Supplementary Fig. 8a** and **Supplementary Fig. 8b**, right). However, given our model selection criteria (**Supplementary Fig. 4a** and **Methods**), some parameters may already correlate intrinsically by model design, since not all parameter combinations generate realistic behavior. Our fitting algorithm might also introduce additional redundancies. Thus, a key prerequisite for any biological interpretation is that correlations observed in real experimental data exceed those expected from model selection and fitting alone. To test this, we developed a statistical pipeline using model-generated behavioral data, applying the same series of steps to synthetic and real data alike.

We first computed Pearson correlations between parameter pairs from the randomly generated models in **Supplementary Fig. 4a**. These are correlations that arise purely from model design and the criteria used to select target models. Comparing pairs of estimated parameter distributions in these synthetic models, we found that most parameters were positively correlated (**Supplementary Fig. 8b**, top left). For example, drift and leak showed a positive relationship, suggesting that only in such configurations does the integrator variable reliably reach threshold at plausible rates. Notably, this does not imply that the parameters are redundant. It simply reflects that certain combinations are more likely to produce realistic model behavior.

We then computed Pearson correlations after fitting these same targets with our optimization method; if substantial new correlations emerged at this stage, it would indicate that our fitting method is not fully able to constrain the parameters, leading to correlated residual errors. The resulting correlation matrix was almost identical to the one obtained before fitting (**Supplementary Fig. 8b**, top middle). Only the diffusion/reset pair showed a strong correlation introduced by the fitting procedure. Given that we can reliably retrieve parameters from model-generated data (**Supplementary Fig. 4b** and **Supplementary Data 1**), the identified interdependence between diffusion and reset after fitting hints to localized covariations of these parameters close to their individual target values. As we are looking for biologically meaningful correlations, we, therefore, excluded this pair from further analysis.

Next, we statistically compared the pre- and post-fitting conditions using a two-sided bootstrap test (**Methods**), quantifying effect size with Cohen's d, the difference between the means of the two bootstrapped distributions, normalized by their pooled standard deviation. We then applied this same comparison to Pearson correlations from real experimental data, allowing us to measure effect strengths between fitted model-generated data and real observations, and to identify correlations exceeding what model design and fitting could explain. We considered a latent parameter correlation biologically significant when it differed significantly from the corresponding correlation in fitted synthetic models. Because this analysis involves comparing multiple parameter pairs and measures correlations between bootstrapped distributions, rather than raw data, we adopted a stricter threshold of $p < 0.01$ to reduce the risk of false positives.

Applying this comparison, we found that several parameter correlations extracted from 5-dpf wild-type zebrafish were significantly stronger than those from fitted model-generated data (**Supplementary Fig. 8b**, bottom right). Specifically, the drift-diffusion, leak-drift, and delay-leak pairs showed significantly elevated correlations. This suggests that larval zebrafish may explicitly link these parameters beyond what chance and model design alone would predict. Repeating this analysis across age groups showed that these relationships remain stable up to 8 dpf (**Supplementary Fig. 8c**).

Together, these results suggest that biological constraints and developmental tuning programs may actively induce specific correlations among the extracted drift-diffusion parameters, dependencies that cannot be explained by model design or fitting artifacts.
